# Supplementary material for: Urinary Antibiotics and Dietary Determinants in Adults in Xinjiang, West China
Source: Nutrients. 2022 Nov 10;14(22):4748. doi: 10.3390/nu14224748 (PMC9692989; doi:10.3390/nu14224748)
Supplement: Supplementary file 1 [file nutrients-14-04748-s001.zip › nutrients-1981167-supplementary.pdf]

Supplementary material

Table S1. The Rate of Detection of Antibiotics in Urine was Related to Selected Dietary Characteristics.

Antibiotics	Overall (n=873)[a]	Pork		Vegetable		Fruits		PDI				uPDI			
		Not eat	Occasionally to eat	Not every day	Every day	Not every day	Every day	Q1	Q2	Q3	Q4	Q1	Q2	Q3	Q4
**Tetracyclines**[†]	128(14.7)	105(14.5)	14(12.8)	2(8.0)	124(15.0)	92(14.9)	35(14.5)	25(2.9)	38(4.4)	41(4.8)	23(2.7)	38(4.4)	39(4.5)	21(2.4)	29(3.4)
chlortetracycline	5(0.6)	3(0.4)	2(1.8)	0(0.0)	5(0.6)	4(0.6)	1(0.4)	0(0.0)	3(0.3)	1(0.1)	1(0.1)	3(0.3)	1(0.1)	0(0.0)	1(0.1)
tetracycline	96(11.0)	76(10.5)	12(11.0)	0(0.0)	95(11.5)	76(12.3)	20(8.3)	17(2.0)	29(3.4)	31(3.6)	19(2.2)	26(3.0)	30(3.5)	15(1.7)	25(2.9)
doxycycline	6(0.7)	5(0.7)	1(0.9）	1(4.0)	5(0.6)	4(0.6)	2(0.8)	2(0.2)	3(0.3)	1(0.1)	0(0.0)	2(0.2)	3(0.3)	1(0.1)	0(0.0)
oxytetracycline[#]	54(6.2)	43(6.0)	7(6.4)	1(4.0)	52(6.3)	31(5.0)	22(9.1)	9(1.0)	15(1.7)	22(2.6)	8(0.8)	17(2.0)	18(2.1)	9(1.0)	9(1.0)
**Fluoroquinolones**[#]	263(30.1)	222(30.7)	28(25.7)	6(24.0)	250(30.2)	196(31.8)	60(24.8)	80(9.3)	60(7.0)	85(9.9)	34(3.9)	72(8.4)	63(7.3)	61(7.1)	63(7.3)
enrofloxacin	12(1.4)	9(1.2)	2(1.8)	0(0.0)	11(1.3)	6(1.0)	5(2.1)	4(0.5)	2(0.2)	4(0.5)	2(0.2)	7(0.8)	1(0.1)	2(0.2)	2(0.2)
norfloxacin[#]	93(10.7)	79(10.9)	10(9.2)	2(8.0)	87(10.5)	78(12.7)	11(4.5)	27(3.1)	19(2.2)	31(3.6)	15(1.7)	21(2.4)	23(2.7)	168(19.5)	184(21.4)
ciprofloxacin[†]	53(6.1)	48(6.6)	4(3.7)	3(12.0)	50(6.0)	38(6.2)	14(5.8)	8(0.9)	16(1.9)	22(2.6)	7(0.8)	14(1.6)	16(1.9)	8(0.9)	15(1.7)
ofloxacin[†‡]	168(19.2)	136(18.8)	22(20.2)	3(12.0)	159(19.2)	124(20.1)	39(16.1)	59(6.9)	38(4.4)	50(5.8)	17(2.0)	43(5.0)	36(4.2)	44(5.1)	41(4.8)
**Macrolides**	61(7.0)	49(6.8)	5(4.6)	3(12.0)	56(6.8)	46(7.5)	14(5.8)	14(1.6)	12(1.4)	25(2.9)	9(1.0)	17(2.0)	17(2.0)	11(1.3)	15(1.7)
azithromycin	33(3.8)	27(3.7)	2(1.8)	2(8.0)	30(3.6)	24(3.9)	9(3.7)	6(0.7)	7(0.8)	13(1.5)	7(0.8)	11(1.3)	8(0.9)	7(0.8)	7(0.8)
clarithromycin	4(0.5)	3(0.4)	0(0.0)	0(0.0)	4(0.5)	2(0.3)	2(0.8)	2(0.2)	0(0.0)	1(0.1)	1(0.1)	1(0.1)	1(0.1)	0(0.0)	2(0.2)
roxithromycin	29(3.3)	24(3.3)	3(2.8)	1(4.0)	27(3.3)	22(3.6)	6(2.5)	6(0.7)	6(0.7)	14(1.6)	2(0.2)	7(0.8)	11(1.3)	4(0.5)	6(0.7)
**Sulfonamides**	78(8.9)	69(9.6)	8(7.3)	3(12.0)	74(8.9)	63(10.2)	16(6.2)	24(2.8)	18(2.1)	21(2.4)	15(1.7)	28(3.3)	24(2.8)	11(1.3)	15(1.7)
sulfamethazine	20(2.3)	19(2.6)	1(0.9)	0(0.0)	19(2.3)	15(2.4)	5(2.1)	9(1.0)	4(0.5)	4(0.5)	3(0.3)	5(0.6)	5(0.6)	2(0.2)	8(0.9)
sulfadiazine	1(0.1)	0(0.0)	1(0.9)	0(0.0)	1(0.1)	1(0.2)	0(0.0)	0(0.0)	1(0.1)	0(0.0)	0(0.0)	0(0.0)	1(0.1)	0(0.0)	0(0.0)
sulfamethoxazole	18(2.1)	14(1.9)	3(2.8)	1(4.0)	17(2.1)	15(2.4)	3(1.2)	4(0.5)	9(1.0)	4(0.5)	1(0.1)	6(0.7)	5(0.6)	4(0.5)	3(0.3)
trimethoprim	58(6.6)	49(6.8)	8(7.3)	3(12.0)	55(6.6)	46(7.5)	12(5.0)	16(1.9)	14(1.6)	15(1.7)	13(1.5)	23(2.7)	19(2.2)	9(1.0)	7(0.8)
**Phenicols**	83(9.5)	72(10.0)	8(7.3)	3(12.0)	79(9.5)	58(9.4)	25(10.3)	32(3.7)	20(2.3)	19(2.2)	12(1.4)	30(3.5)	22(2.6)	21(2.4)	10(1.2)
chloramphenicol[†]	51(5.8)	48(6.4)	3(2.8)	2(8.0)	49(5.9)	33(5.4)	18(7.4)	23(2.7)	12(1.4)	12(1.4)	4(0.5)	19(2.2)	15(1.7)	11(1.3)	6(0.7)
florfenicol	33(3.8)	27(3.7)	5(4.6)	1(4.0)	32(3.9)	26(4.2)	7(2.9)	11(1.3)	9(1.0)	6(0.7)	7(0.8)	10(1.2)	9(1.0)	9(1.0)	5(0.6)
thiamphenicol[*]	5(0.6)	2(0.3)	3(2.8)	0(0.0)	4(0.5)	4(0.6)	1(0.4)	1(0.1)	1(0.1)	1(0.1)	2(0.2)	1(0.1)	1(0.1)	3(0.3)	0(0.0)

[a] Frequency of positive detection (%).

[*] $P<0.05$ vs Pork. [#] $P<0.05$ vs Fruits. [†] $P<0.05$ vs PDI. [‡] $P<0.05$ vs uPDI.

Table S2. Detection Rates of Urinary Antibiotics in Relation to Selected Demographic Characteristics.

Antibiotics	Overall (n=873)[a]	Age				Education				Monthly expenditure per capita （RMB）		
		35-45	46-55	56-65	66-75	＜Primary	Primary	Secondary	≥High school	≤240	240-333.33	＞333.33
**Tetracyclines**	128(14.7)	27(11.4)	43(14.0)	43(18.9)	15(14.7)	37(16.7)	53(13.8)	33(15.9)	5(8.5)	38(17.4)	24(14.5)	64(13.4)
chlortetracycline	5(0.6)	0(0.0)	2(0.7)	2(0.9)	1(1.0)	1(0.5)	2(0.5)	1(0.5)	1(1.7)	1(0.5)	1(0.6)	3(0.6)
tetracycline	96(11.0)	18(7.6)	35(11.4)	32(14.0)	11(10.8)	27(12.2)	42(10.9)	22(10.6)	5(8.5)	28(12.8)	18(10.9)	50(10.4)
doxycycline	6(0.7)	1(0.4)	2(0.7)	3(1.3)	0(0.0)	0(0.0)	5(1.3)	1(0.5)	0(0.0)	2(0.9)	1(0.6)	3(0.6)
oxytetracycline	54(6.2)	11(4.7)	16(5.2)	21(9.2)	6(5.9)	18(8.6)	19(4.9)	14(6.7)	2(3.4)	17(7.8)	9(5.5)	26(5.4)
**Fluoroquinolones**[*†]	263(30.1)	61(25.8)	80(26.1)	83(36.4)	39(38.2)	80(36.2)	117(30.4)	55(26.4)	11(18.6)	68(31.2)	53(32.1)	139(29)
enrofloxacin	12(1.4)	1(0.4)	7(2.3)	1(0.4)	3(2.9)	1(0.5)	6(1.6)	4(1.9)	1(1.7)	2(0.9)	3(1.8)	6(1.3)
norfloxacin[*†]	93(10.7)	17(7.2)	27(8.8)	35(15.4)	14(13.7)	33(14.9)	42(10.9)	16(7.7)	2(3.4)	25(11.5)	21(12.7)	46(9.6)
ciprofloxacin	53(6.1)	13(5.5)	15(4.9)	17(7.5)	8(7.8)	18(8.1)	20(5.2)	10(4.8)	5(8.5)	17(7.8)	8(4.8)	27(5.6)
ofloxacin	168(19.2)	43(18.2)	54(17.6)	48(21.1)	23(22.5)	40(18.1)	82(21.3)	39(18.8)	7(11.9)	40(18.3)	35(21.2)	91(19.0)
**Macrolides**	61(7.0)	25(10.6)	16(5.2)	14(6.1)	6(5.9)	16(7.2)	30(7.8)	10(4.8)	5(8.5)	13(6.0)	11(6.7)	35(7.3)
azithromycin[*]	33(3.8)	16(6.8)	5(1.6)	9(3.9)	3(2.9)	9(4.1)	16(4.2)	4(1.9)	4(6.8)	7(3.2)	5(3.0)	20(4.2)
clarithromycin	4(0.5)	1(0.4)	2(0.7)	1(0.4)	0(0.0)	1(0.5)	2(0.5)	1(0.5)	0(0.0)	1(0.5)	1(0.6)	2(0.4)
roxithromycin	29(3.3)	11(4.7)	10(3.3)	5(2.2)	3(2.9)	8(3.6)	12(3.1)	7(3.4)	2(3.4)	7(3.2)	5(3.0)	16(3.3)
**Sulfonamides**	78(8.9)	20(8.5)	25(8.1)	27(11.8)	6(5.9)	24(10.9)	38(9.9)	10(4.8)	6(10.2)	19(8.7)	13(7.9)	46(9.6)
sulfamethazine	20(2.3)	4(1.7)	9(2.9)	7(3.1)	0(0.0)	6(2.7)	10(2.6)	1(0.5)	3(5.1)	7(3.2)	3(1.8)	10(2.1)
sulfadiazine	1(0.1)	0(0.0)	1(0.3)	0(0.0)	0(0.0)	0(0.0)	1(0.3)	0(0.0)	0(0.0)	0(0.0)	0(0.0)	1(0.2)
sulfamethoxazole	18(2.1)	4(1.7)	3(1.0)	10(4.4)	1(1.0)	4(1.8)	10(2.6)	1(0.5)	3(5.1)	3(1.4)	2(1.2)	13(2.7)
trimethoprim	58(6.6)	17(7.2)	17(5.5)	18(7.9)	6(5.9)	18(8.1)	29(7.5)	9(4.3)	2(3.4)	12(5.5)	10(6.1)	36(7.5)
**Phenicols**	83(9.5)	23(9.7)	27(8.8)	22(9.6)	11(10.8)	22(10.0)	46(11.9)	12(5.8)	3(5.1)	21(9.6)	15(9.1)	47(9.8)
chloramphenicol[†]	51(5.8)	14(5.9)	17(5.5)	13(5.7)	7(6.9)	15(6.8)	30(7.8)	6(2.9)	0(0.0)	16(7.3)	6(3.6)	29(6.1)
florfenicol	33(3.8)	9(3.8)	12(3.9)	8(3.5)	4(3.9)	6(2.7)	17(4.4)	7(3.4)	3(5.1)	4(1.8)	9(5.5)	20(4.2)
thiamphenicol	5(0.6)	0(0.0)	3(1.0)	2(0.9)	0(0.0)	2(0.9)	2(0.5)	1(0.5)	0(0.0)	1(0.5)	0(0.0)	4(0.8)

a Frequency of positive detection (%).

[*] P<0.05 vs Age. [†] P<0.05 vs Education.

Table S3. Concentration (ng/mL) of Urinary Antibiotics in Relation to Dietary Characteristics.

Antibiotics	Overall (n=873)[a,b]	Pork[a,b]		Vegetable[a,b]		Fruits[a,b]		PDI [a,b]				uPDI [a,b]			
		Not eat	Occasionally to eat	Not every day	Every day	Not every day	Every day	Q1	Q2	Q3	Q4	Q1	Q2	Q3	Q4
**Tetracyclines†**	17	18	16	13	18	18	18	5.2	71	23	14	23	21	12	14
chlortetracycline	0.1	0.1	0.1	0.1	0.1	0.1	0.1	0.1	0.1	0.1	0.1	0.1	0.1	0.1	0.1
tetracycline	9	9	7.2	0.2	10	9.7	8.8	4.2	21	11	9.7	19	7.3	4.3	12
doxycycline^	0.1	0.1	0.1	11	0.1	0.1	0.1	0.1	0.1	0.1	0.1	0.1	0.1	0.1	0.1
oxytetracycline#	1.5	1.6	1.2	4.2	1.7	0.8	3.5	0.1	4.3	3.2	0.1	1.4	4.3	0.7	0.1
**Fluoroquinolones†**	7.7	8.3	2.3	180	7.5	8.1	6.1	8	8	8	5	8	3.1	8.5	11
enrofloxacin	0.1	0.1	0.1	0.1	0.1	0.1	0.1	0.1	0.1	0.1	0.1	0.1	0.1	0.1	0.1
norfloxacin#	1	1	0.7	13	1	1.1	0.1	1.1	1	1.5	0.5	0.8	0.7	1.1	3
ciprofloxacin†	0.8	0.7	0.1	180	0.6	0.6	0.7	0.1	0.8	0.9	0.1	0.3	0.6	0	0.8
ofloxacin†‡	1.6	1.8	1.1	2.4	1.6	1.5	1.8	1.9	2.2	2.5	0.6	1.9	1.2	1.4	2.3
**Macrolides**	2.1	2.2	0.1	9.5	2	2. 9	1	1.4	1.1	23	1.3	0.9	3.9	2	22
azithromycin	0.1	0.1	0.1	1.5	1	1	1	0.1	0.1	0.9	0.1	0.1	0.1	0.1	0.1
clarithromycin	0.02	0.02	0.02	0.02	0.02	0.02	0.02	0.02	0.02	0.02	0.02	0.02	0.02	0.02	0.02
roxithromycin	0.2	0.2	0.2	9	0.2	0.2	0.2	0.2	0.2	0.9	0.2	0.2	0.2	0.2	0.2
**Sulfonamides**	0.3	0.3	0.2	1. 9	0.3	0.3	0.1	0.3	1.1	0.2	0.2	0.3	0.2	0.1	0.3
sulfamethazine	0.1	0.1	0.1	0.1	0.1	0.1	0.1	0.1	0.1	0.1	0.1	0.1	0.1	0.1	0.1
sulfadiazine*	0.2	0.2	0.2	0.2	0.2	0.2	0.2	0.2	0.2	0.2	0.2	0.2	0.2	0.2	0.2
sulfamethoxazole†	0.1	0.1	0.1	1.7	0.1	0.1	0.1	0.1	0.01	0.1	0.1	0.1	0.1	0.1	0.1
trimethoprim	0.1	0.1	0.2	0.5	0.1	0.2	0.03	0.1	0.7	0.1	0.2	0.2	0.2	0.1	0.1
**Phenicols**	0.1	0.1	0.1	0.1	0.1	0.1	0.1	0.1	0.1	0.1	0.1	0.1	0.1	0.1	0.01
chloramphenicol†	0.04	0.04	0.1	0.1	0.04	0.02	0.1	0.1	0.1	0.1	0.1	0.1	0.04	0.04	0.1
florfenicol	0.1	0.1	0.01	0.11	0.1	0.1	0.1	0.1	0	0.1	0.1	0.1	0.1	0.04	0.1
thiamphenicol*	0.1	0.1	0.1	0.1	0.1	0.1	0.1	0.1	0.1	0.1	0.1	0.1	0.1	0.1	0.1

[a] The 95th percentile urinary concentration (ng/mL).

[b] Antibiotic concentrations below LOD were replaced with those of LOD/2.

* P<0.05 vs Pork. ^ P<0.05 vs Vegetable. # P<0.05 vs Fruits. † P<0.05 vs PDI. ‡ P<0.05 vs uPDI.

Table S4. Concentration (ng/mL) of Urinary Antibiotics in Relation to Selected Demographic Characteristics.

Antibiotics	Overall (n=873)[a,b]	Age[a,b]				Education[a,b]				Monthly expenditure per capita (RMB)[a,b]		
		35-45	46-55	56-65	66-75	＜Primary	Primary	Secondary	≥High school	≤240	240-333.33	＞333.33
**Tetracyclines**	17	14	12	53	8.2	27	16	8.9	91	23	12	18
chlortetracycline	0.1	0.1	0.1	0.1	0.1	0.1	0.1	0.1	0.1	0.1	0.1	0.1
tetracycline	9	7	7.5	40	7.6	20	11	5.4	15	11	6. 8	12
doxycycline	0.1	0.1	0.1	0.1	0.1	0.1	0.1	0.1	0.1	0.1	0.1	0.1
oxytetracycline	1.5	0.1	1	3.7	2.5	3.5	0.5	1.9	0.1	3.8	2.1	1
**Fluoroquinolones***^	7.7	4	4	18	9.3	11	8	7.2	10	4.8	12	7.1
enrofloxacin	0.1	0.1	0.1	0.1	0.1	0.1	0.1	0.1	0.1	0.1	0.1	0.1
norfloxacin*^	1	0.5	1	3.6	1.4	1.4	1.4	0.6	0.1	1.1	1.4	1
ciprofloxacin	0.6	0.5	0.1	0.7	0.7	0.8	0.3	0.3	4	0.6	0.2	0.5
ofloxacin	1.6	1.3	1	2.4	2.7	1.1	2.2	3.1	2.1	1.3	4. 8	1.1
**Macrolides**	2.1	54	1.2	1.1	2.2	4.2	4.6	0.5	13	1.3	3.3	2.4
azithromycin*	0.1	2.3	0.1	0.1	0.1	0.1	0.1	0.1	1.9	0.1	0.1	0.1
clarithromycin	0.02	0.02	0.02	0.02	0.02	0.02	0.02	0.02	0.02	0.02	0.02	0.02
roxithromycin	0.2	0.1	0.2	0.2	0.2	0.2	0.2	0.2	0.2	0.2	0.2	0.2
**Sulfonamides**	0.3	0.3	0.2	0.3	0.2	0.2	0.3	0.02	0.6	0.3	0.3	0.3
sulfamethazine	0.1	0.1	0.1	0.1	0.1	0.1	0.1	0.1	0.3	0.1	0.1	0.1
sulfadiazine	0.2	0.2	0.2	0.2	0.2	0.2	0.2	0.2	0.2	0.2	0.2	0.2
sulfamethoxazole*	0.1	0.1	0.1	0.1	0.1	0.1	0.1	0.1	0.3	0.1	0.1	0.1
trimethoprim	0.2	0.2	0.1	0.2	0.2	0.2	0.1	0.1	0.1	0.1	0.1	0.2
**Phenicols**	0.1	0.1	0.1	0.1	0.2	0.1	0.1	0.1	0.1	0.1	0.1	0.1
chloramphenicol^	0.04	0.04	0.02	0.04	0.1	0.04	0.1	0.1	0.1	0.04	0.1	0.04
florfenicol	0.1	0.1	0.1	0.1	0.1	0.1	0.1	0.1	0.1	0.1	0.04	0.1
thiamphenicol	0.1	0.1	0.1	0.1	0.1	0.1	0.1	0.1	0.1	0.1	0.1	0.1

[a] The 95th percentile urinary concentration (ng/mL).

[b] Antibiotic concentrations below LOD were replaced with those of LOD/2.

* P<0.05 vs Age. ^ P<0.05 vs Education.

Table S5. Independent Variables Associated with the Detection of Doxycycline, Oxytetracycline, Fluoroquinolones, Norfloxacin, Azithromycin, Chloramphenicol and Thiamphenicol in urine were Analyzed using Multivariate Binary Logistic Regression.

**Type of participants**<br>**Variable**	**doxycycline**[a]	**oxytetracycline**[a]	**fluoroquinolones**[a]	**norfloxacin**[a]	**azithromycin**[a]	**chloramphenicol**[a]	**thiamphenicol**[a]
Not to eat pork	Ref.	Ref.	Ref.	Ref.	Ref.	Ref.	Ref.
Eat pork occasionally	1.6(0.2-14)	1.04(0.4-2.5)	0.7(0.4-1.2)	0.7(0.3-1.4)	0.7(0.2-3)	0.6(0.2-2)	**15(1.7-131)***
Not to eat fruit every day	Ref.	Ref.	Ref.	Ref.	Ref.	Ref.	Ref.
Eat fruit every day	1.4(0.2-8.9)	1.7(0.9-3.3)	0.8(0.6-1.2)	**0.4(0.2-0.7)***	0.6(0.3-1.6)	1.5(0.8-3)	1.1(0.1-12)
PDI(Q1)	Ref.	Ref.	Ref.	Ref.	Ref.	Ref.	Ref.
PDI(Q2)	1.9(0.3-12)	2.2(0.9-5.3)	1(0.6-1.5)	1.1(0.6-2.1)	1.3(0.4-4.2)	0.6(0.3-1.3)	1.2(0.1-22)
PDI(Q3)	0.5(0.4-5.4)	2.1(0.9-4.7)	1.2(0.8-1.7)	1.5(0.8-2.6)	1.9(0.7-5.4)	**0.4(0.2-0.9)***	1.2(0.1-22)
PDI(Q4)	——	1.1(0.4-3.1)	**0.6(0.4-0.99)***	1.2(0.6-2.3)	1.8(0.5-5.8)	**0.2(0.1-0.6)***	3.2(0.2-42)
uPDI(Q1)	Ref.	Ref.	Ref.	Ref.	Ref.	Ref.	Ref.
uPDI(Q2)	1.7(0.3-11)	1.6(0.8-3.4)	1.1(0.7-1.6)	1.2(0.6-2.3)	0.8(0.3-2.2)	0.9(0.4-1.9)	1(0.1-18)
uPDI(Q3)	7.3(0.1-8.8)	1.1(0.5-2.7)	1.6(1-2.4)	1.6(0.8-3)	0.8(0.3-2.3)	0.9(0.4-2.1)	4.7(0.4-54)
uPDI(Q4)	——	1(0.4-2.4)	1.1(0.7-1.8)	1.3(0.7-2.5)	0.7(0.2-2.2)	0.4(0.2-1.2)	——

[a] Odds ratio (OR) and 95%CI.

Notes: All Models were adjusted for age and education

Statistical significant(*P*-values＜0.05)estimates are indicated in *

Table S6. Independent Variables Associated with the Concentration of Fluoroquinolones using Multivariate Multinomial Logistic Regression.

Variable	Tier 2[a,b,c]	Tier 3[a,b,c]
**Type of participants**		
PDI(Q1)	Ref.	Ref.
PDI(Q2)	1.7(0.9-3.2)	**2.4(1.2-4.7)***
PDI(Q3)	1.7(0.9-3.3)	1.6(0.8-3.3)
PDI(Q4)	1.5(0.8-2.9)	1.8(0.9-3.6)
uPDI(Q1)	Ref.	Ref.
uPDI(Q2)	1.4(0.7-2.4)	1(0.5-1.9)
uPDI(Q3)	1.1(0.6-1.9)	0.7(0.4-1.2)
uPDI(Q4)	0.7(0.4-1.2)	1(0.6-1.6)

[a] Tier 1 (reference): concentrations lower than LOD; Tier 2: LOD-median of detected concentrations; Tier 3: higher than the median of detected concentrations.

[b] Odds ratio (OR) and 95%CI.

[c] All Models were adjusted for age.

Statistical significant(*P*-values$<$0.05)estimates are indicated in *

Table S7. Independent Variables Associated with the Concentration of Norfloxacin using Multivariate Multinomial Logistic Regression.

Variable	Tier 2[a,b,c]	Tier 3[a,b,c]
**Type of participants**		
Not to eat fruit every day	Ref.	Ref.
Eat fruit every day	**2.6(1.1-6.2)***	**3.1(1.2-7.9)***

[a] Tier 1 (reference): concentrations lower than LOD; Tier 2: LOD-median of detected concentrations; Tier 3: higher than the median of detected concentrations.

[b] Odds ratio (OR) and 95%CI.

[c] All Models were adjusted for age.

Statistical significant(*P*-values$<$0.05)estimates are indicated in *
